# A prognostic mathematical model based on tumor microenvironment-related genes expression for breast cancer patients

**DOI:** 10.3389/fonc.2023.1209707

**Published:** 2023-10-04

**Authors:** Hong Chen, Shan Wang, Yuting Zhang, Xue Gao, Yufu Guan, Nan Wu, Xinyi Wang, Tianyang Zhou, Ying Zhang, Di Cui, Mijia Wang, Dianlong Zhang, Jia Wang

**Affiliations:** ^1^ Department of Breast Surgery, Second Affiliated Hospital of Dalian Medical University, Dalian, China; ^2^ Department of Pathology, The First Affiliated Hospital of Dalian Medical University, Dalian, China; ^3^ Department of Breast and Thyroid Surgery, Affiliated Zhongshan Hospital of Dalian University, Dalian, China; ^4^ Information Center, Second Affiliated Hospital of Dalian Medical University, Dalian, China

**Keywords:** breast cancer, tumor microenvironment, prognostic, resistance, therapeutic sensitivity

## Abstract

**Background:**

Tumor microenvironment (TME) status is closely related to breast cancer (BC) prognosis and systemic therapeutic effects. However, to date studies have not considered the interactions of immune and stromal cells at the gene expression level in BC as a whole. Herein, we constructed a predictive model, for adjuvant decision-making, by mining TME molecular expression information related to BC patient prognosis and drug treatment sensitivity.

**Methods:**

Clinical information and gene expression profiles were extracted from The Cancer Genome Atlas (TCGA), with patients divided into high- and low-score groups according to immune/stromal scores. TME-related prognostic genes were identified using Kaplan-Meier analysis, functional enrichment analysis, and protein-protein interaction (PPI) networks, and validated in the Gene Expression Omnibus (GEO) database. Least absolute shrinkage and selection operator (LASSO) Cox regression analysis was used to construct and verify a prognostic model based on TME-related genes. In addition, the patients’ response to chemotherapy and immunotherapy was assessed by survival outcome and immunohistochemistry (IPS). Immunohistochemistry (IHC) staining laid a solid foundation for exploring the value of novel therapeutic target genes.

**Results:**

By dividing patients into low- and high-risk groups, a significant distinction in overall survival was found (p < 0.05). The risk model was independent of multiple clinicopathological parameters and accurately predicted prognosis in BC patients (p < 0.05). The nomogram-integrated risk score had high prediction accuracy and applicability, when compared with simple clinicopathological features. As predicted by the risk model, regardless of the chemotherapy regimen, the survival advantage of the low-risk group was evident in those patients receiving chemotherapy (p < 0.05). However, in patients receiving anthracycline (A) therapy, outcomes were not significantly different when compared with those receiving no-A therapy (p = 0.24), suggesting these patients may omit from A-containing adjuvant chemotherapy. Our risk model also effectively predicted tumor mutation burden (TMB) and immunotherapy efficacy in BC patients (p < 0.05).

**Conclusion:**

The prognostic score model based on TME-related genes effectively predicted prognosis and chemotherapy effects in BC patients. The model provides a theoretical basis for novel driver-gene discover in BC and guides the decision-making for the adjuvant treatment of early breast cancer (eBC).

## 1 Introduction

Breast cancer (BC) is the most common malignancy in women. According to cancer burden data from the International Agency for Research on Cancer (World Health Organization, 2020), up to 2.26 million new BC cases were recorded globally, and together with lung and colorectal cancer, accounts for more than half of new female cancers (1). Long-term survival in BC patients varies with the stage status at the time of initial diagnosis. The overall 5-year BC survival rate is 98% for stage I, 92% for stage II, 75% for stage III, and a sudden drop to 27% for stage IV (2). Currently, the main BC treatments include surgery, radiotherapy, and systemic therapy (chemotherapy, endocrine therapy, and targeted medication) (3–6). However, 40% of BC patients are resistant to current available chemotherapy or targeted therapies (7). With the high heterogeneity of BC, the traditional immunohistochemical staining quadruple type is no longer able to provide more accurate personalized treatment for early BC (eBC) patients, especially considering the impact of new targets and targeted drugs. Multigene panels, such as PAM50 intrinsic BC subtypes, 21 Gene Recurrence Score and 70-gene Prognostic Signature have quietly stepped on to the historical stage, were incorporated into the TNM staging system by the American Joint Committee on Cancer (8^th^ edition) (8). Unequivocally, for prognosis predictions, multivariable indicators are more accurate and objective when compared with single biomarkers (9). Hence, to identify more biomarkers and guide precise personalized eBC treatment, more risk models based on gene expression profiles, are required.

Tumor progression is a complex process with interactions occurring among tumor cells, the tumor microenvironment (TME), and the immune system (10–12). The TME reflects the cellular environment of the tumor (13, 14), including cell components other than tumor cells, e.g., immune and stromal cells, extracellular matrix molecules, and cytokines (15, 16). Previous studies indicated that stromal cells have important roles in tumor growth, disease development (17, 18), and drug resistance (19). Immune cells exert regulatory and destructive effects toward tumor cells and may have dual promotional and antagonistic functions (20–22). Through crosstalk, they participate in tumor processes and development, are involved in mechanisms underpinning the TME, and contribute to tumor diagnostic and prognostic evaluation (23–26). Increasingly, the TME is considered a therapy target (27, 28); the prediction and prognostic value of tumor-infiltrating lymphocytes (TILs) in BC is gradually being recognized (29, 30). For example, ECOG2197 and ECOG1199 clinical studies identified an approximate 15% reduction in relapse and mortality rates for every 10% increase in TIL levels (30). The KEYNOTE-086 study indicated that higher TIL levels were associated with significant improvements in objective response rates for pembrolizumab (31). However, few studies have reported on how the TME may be used as a prognostic and predictive biomarker in assessing tumor immunity and treatment efficiency in BC patients. In our study, we show that TME may be used to accurately predict the prognosis in BC patients, independent of multiple clinicopathological factors, and predict the efficacy of chemotherapy and immunotherapy in these patients. Critically, low-risk patients in our prediction model may be exempted from the A-adjuvant chemotherapy regimens, thus providing guidance for patients with de-escalated individual treatment.

Yoshihara et al. developed the ESTIMATE algorithm where gene expression profiles were used to predict infiltrating stromal and immune cell levels in the TME (23). Previous studies reported the algorithm was effective in predicting TME status, with immune and stromal scores predicting tumor-associated normal cells penetration. However, studies focused exclusively on immune cells (32, 33) rather than stromal cells, and largely ignored their role in tumorigenesis and development. Secondly, due to complex reticular regulatory mechanisms in the TME, a single pathway or single cell subpopulation cannot fully identify mechanisms between the TME and tumors (34). Therefore, a comprehensive understanding of tumor-associated normal cells in tumor tissues may provide important insights into BC biology. In our study, we comprehensively evaluated molecular expression networks in stromal and immune cells to (1) understand the significance of TME-related genes and (2) provide a more accurate and comprehensive assessment of the TME during BC development and treatment.

We used several bioinformatics approaches to explore the TME during BC occurrence and progression. Based on TME-related genes expression, we constructed a new prognostic risk model to evaluate the prognostic value of the TME. Differences between the immune microenvironment in BC patients were comprehensively analyzed. Additionally, underlying signal pathways were preliminarily elucidated. This work provides new insights into the molecular mechanisms underpinning BC tumor occurrence and development, and may help predict prognosis in BC patients and assess therapeutic efficacy.

## 2 Methods

### 2.1 Clinical specimens

Two BC tissue specimens were obtained from patients at the Second Hospital of Dalian Medical University. Invasive breast cancer was pathologically confirmed in all patients not on chemotherapy or radiotherapy before tissue collection. Written informed consent was obtained from patients, and the study was approved by the Ethics and Human Subject Committee of the Second Hospital of Dalian Medical University (NO.2023191). Procedures were performed according to hospital guidelines and regulations.

### 2.2 Data sources

Gene expression matrices of enrolled patients were obtained from The Cancer Genome Atlas (TCGA) and the Gene Expression Omnibus (GEO) databases. We included 1,069 BC samples from TCGA as the training cohort. The gene-expression profiles of TCGA-BRCA in the Fragments Per Kilobase per Million (FPKM) format were obtained from the TCGA portal (http://cancergenome.nih.gov), and then the ID conversion was carried out through the operation of ENSG ID to GeneSymbol, and finally the data standardization was carried out, and the standardization method is log2 (X+1). In addition, the BC patients’ clinical data (gender, age, histological type, and survival) were downloaded from TCGA. After searching the datasets with more than 150 human breast cancer samples with complete expression profile data, we selected the GSE42568, GSE88770, GSE48390, and GSE162228 dataset from the GEO as the validation cohort. These datasets were verified using the GPL570 platform. To ensure the scientificity and accuracy of the research, we successfully removed batch effect with COMBAT when combining GEO multi-data sets (**Supplementary Figure S1**). Additionally, clinical survival and outcome data of BC patients were also downloaded from this database.

### 2.3 Identifying differentially expressed genes (DEGs)

Data analysis was performed using the “limma” R package. Fold change > 1.5, p < 0.05, and false discovery rate (FDR) < 0.05 were set as the cutoffs to screen for DEGs.

### 2.4 DEG enrichment analysis

Gene ontology (GO) and Kyoto Encyclopedia of Genes and Genomes (KEGG) analyses were performed to enrich the DEGs into associated pathways using the “clusterProfiler” R package (version 3.14.3). p < 0.05 and FDR < 0.05 were considered significant.

### 2.5 Constructing and validating a risk model based on TME-related genes

Least absolute shrinkage and selection operator (LASSO) Cox regression analysis identified genes most correlated with OS, and 10-round cross-validation was performed to prevent overfitting. The risk score for each patient was then calculated based on the expression levels of genes. Risk score: -0.0419970982477039 * NPY1R - 0.162055812415471 * CELSR2 - 0.043004672153174 * STC2 - 0.0716026845406244 * SCUBE2 + 0.2810654696502 * GIMAP2 + 0.0773881988402307 * HLA-DPB1 - 0.0232515777318596 * CXCL14 - 0.721867840891611 * KLRB1 - 0.253187064109637 * BIRC3 - 0.0587584464454724 * IL18 - 0.242105852075788 * PSMB8 + 0.198881881356143 * CD1C + 0.0814403392760682 * TNFAIP8 + 0.076656198308623 * IRF1. According to the median risk score, BC patients were divided into high- and low-risk groups. Kaplan–Meier analysis was employed to estimate the difference in OS between the categorized patients via the R package “survival.” The prognostic capability of the risk model was validated using time- dependent receiver operating characteristic (ROC) analysis with the R package “pROC”.

### 2.6 Evaluation of risk model independence

Univariate and multivariable Cox regression analyses were performed to estimate whether the risk score was an independent predictor of BC prognosis. A subgroup analysis was conducted to confirm the independence of the risk model. The patients with BC in the training cohort were regrouped into new subgroups based on different clinical characteristics, and the patients in each subgroup were stratified into high- and low-risk groups, based on the median risk score.

### 2.7 Immunohistochemistry (IHC)

Patient tissue specimens were fixed in 10% neutral formalin, embedded in paraffin, and sectioned into 4 µm sections before staining. Sections were deparaffinized, rehydrated, and blocked for endogenous peroxidase activity. Next, antigen retrieval was performed in citrate buffer (pH 6.0) and sections autoclaved for 90 s at 121°C. After washing in phosphate buffered saline (3 min × 3), sections were blocked in goat serum at room temperature for 30 min and incubated with primary antibodies (PSMB8, (1:200), Proteintech Group, IL, USA; cIAP2, (1:200), Proteintech Group, IL, USA) overnight at 4° C. The next day, sections were incubated with secondary antibodies (Maxin Biotechnologies, China) and treated with diaminobenzidine hydrochloride to visualize immunoreactivity. The immunohistochemical scoring was performed independently by two experienced pathologists, who had no knowledge of the clinicopathological information.

### 2.8 Nomogram construction

Nomograms are user-friendly clinical tools used to predict disease prognosis. The risk score and clinical parameters were subjected to univariate Cox regression analysis, and features with P values < 0.05 were subjected to multivariable COX regression analysis. Features with p values < 0.05 after multivariate analysis were incorporated into nomograms that were constructed to predict the 3- and 5-year OS rates. The nomogram was based on three independent prognostic factors: age, tumor stage, and the risk score. Factors corresponded to a specific point by drawing a line straight up to the point axis. The sum of the three factor points indicated the total points. By drawing a perpendicular line from the total point axis to the two-outcome axes, estimated 3- and 5-year OS probabilities were obtained. Observed 3- and 5-year OS rates were compared with predicted rates to further verify predictive performance. We assessed nomogram goodness-of-fit using calibration plots.

### 2.9 Immune analysis

The estimation of stromal and immune cells in malignant tumor tissues using expression data (ESTIMATE) method was applied to calculate the immune score, stromal score, and ESTIMATE score of the patients, via the R package “estimate”. Tumor immune estimation resource (TIMER) analysis was conducted to evaluate the abundance of six types of immune cells (neutrophils, CD4 T cells, macrophages, CD8 T cells, dendritic cells (DCs), and B cells). The MCPcounter (microenvironment cell populations-counter) algorithm was also used to assess T cell, CD8 T cell, cytotoxic lymphocyte, B cell lineage, natural killer (NK) cell, monocytic cell lineage, myeloid DC, neutrophil, endothelial cell, and fibroblast abundance.

### 2.10 Immune infiltration analysis of hub genes

TIMER was used to analyze correlations between hub gene expression and the degree of lymphocyte infiltration. TISIDB was also used to analyze correlations between hub gene expression and immune molecule expression in BC. We used the GSCA Lite (A Web Server for Gene Set Cancer Analysis: http://bioinfo.life.hust.edu.cn/web/GSCALite) online tool to analyze the correlation between hub genes expression and sensitivity to current chemotherapeutic or targeted drugs for BC.

### 2.11 Statistical analysis

Statistical analyses were completed using R (version 3.6.3). Discontinuous data were presented as number (percentage), and continuous data were displayed as mean± standard deviation. The Wilcoxon rank sum test was utilized to compare two groups and the Kruskal-Wallis test to compare multiple groups. In addition, the survfit function of “survival” package in R was used to analyze the prognostic differences between the two groups, and the log-rank test was used to further evaluate the significance of prognostic differences between the two groups. Statistical significance was defined as p < 0.05.

## 3 Results

### 3.1 Immune scores and stromal scores are significantly associated with BC subtypes, hormone receptor status, and overall survival (OS)

We downloaded the gene expression profiles and clinical information of 1,069 BC patients from The Cancer Genome Atlas (TCGA). Based on gene expression, BC can be mainly classified into Luminal A, Luminal B, HER2-enriched, Basal-like, and Normal-like (35, 36). The ESTIMATE algorithm showed that the highest mean immune score of Normal-like subtype was highest among all five subtypes, followed by Basal-like subtype, HER2-enriched subtype, and Luminal A subtype. The Luminal B subtype cases had the lowest immune scores (**Supplementary Figure S2A**, p < 0.0001). However, stromal scores, from high to low, were Normal-like, Luminal A, HER2-enriched, Luminal B, and Basal-like (**Supplementary Figure S2B**, p < 0.0001). The mammary gland is a hormone-responsive organ- the endocrine system is closely related to its development and disease occurrence, therefore we performed correlation analyses between immune and stromal scores and hormone receptor status. As shown in **Supplementary Figure S2C**, patients with progesterone receptor positive (PR+) had lower immune scores when compared with progesterone receptor negative (PR-) patients (p < 0.01), while estrogen receptor positive (ER+) patients had lower scores when compared with estrogen receptor negative (ER-) patients (p < 0.0001). In contrast, PR+/ER+ patients had higher scores when compared with PR-/ER- patients, and ER+ patients had higher when compared with ER- patients in the stromal scores (**Supplementary Figure S2D**, p < 0.0001). Thus, stromal and immune scores were significantly associated with BC subtypes and hormone receptor status.

To identify potential OS correlations with immune scores and/or stromal scores, we divided our cohort into top and bottom halves (high vs. low score groups) based on their scores. Kaplan-Meier survival curves showed that median OS in the low score group was longer when compared with the high score group when based on immune scores (**Supplementary Figure S2E**, p = 0.01). Consistently, patients with lower stromal scores had longer median OS when compared with patients with higher stromal scores (**Supplementary Figure S2F**, p = 0.85), although statistical significance was not observed.

### 3.2 Differentially expressed genes (DEGs) in BC and correlations with OS

**Figure 1:**
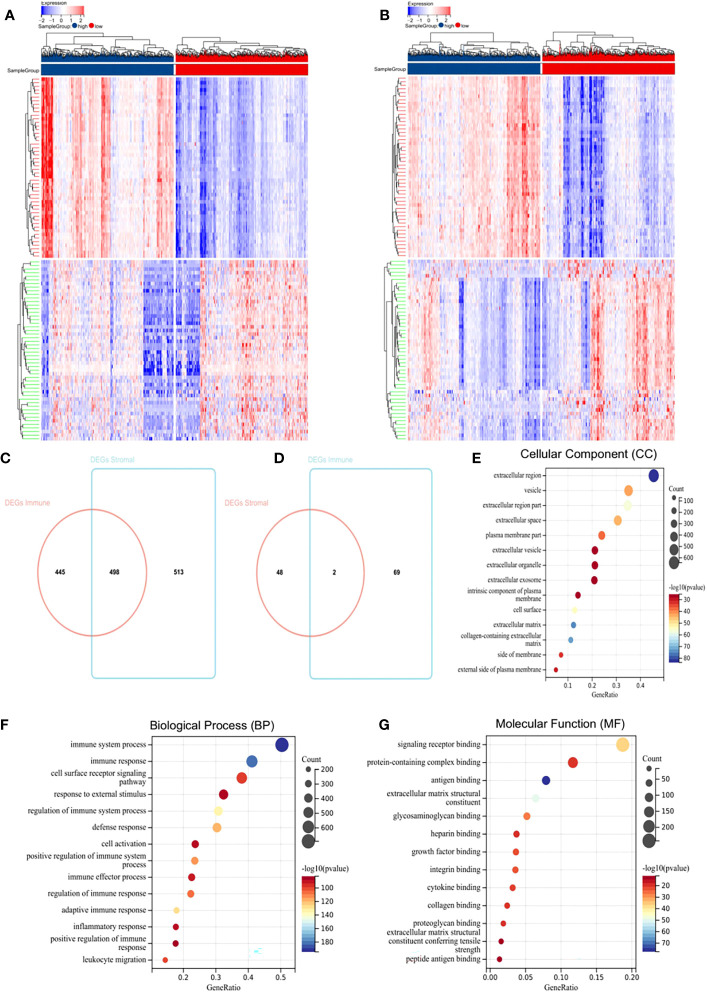
Differentially expressed genes (DEGs) in BC and their correlations with overall survival (OS). **(A)** Heatmap of the DEGs of immune scores. **(B)** Heatmap of the DEGs of stromal scores. **(C, D)** Venn diagrams showing the number of commonly upregulated **(C)** or downregulated **(D)** DEGs in stromal and immune score groups. **(E–G)** Gene Ontology analysis (GO) analysis.

To determine global gene expression profile correlations with immune scores and/or stromal scores, we compared Affymetrix microarray data in 1,069 BC patients. Heatmaps in **Figure 1** showed distinct gene expression profiles of cases belong to immune scores/stromal scores groups. Based on immune scores, 943 genes were upregulated, and 71 genes downregulated in the high score group than the low score group (**Figure 1A**, fold change > 1.5, p < 0.05). Similarly, 1,011 genes were upregulated, and 50 genes were downregulated in the high score group (**Figure 1B**, fold change > 1.5, p < 0.05). Moreover, Venn diagrams (**Figures 1C, D**) showed that 498 genes were upregulated in the high-score group, while two genes were downregulated. We performed subsequent analyses by focusing on all DEGs obtained based on comparisons of immune and stromal scores. To determine potential DEGs functions, we performed functional enrichment analysis on 1,574 DEGs. Top Gene Ontology (GO) terms included immune system process, immune response, extracellular matrix, signalling receptor binding, and integrin binding (**Figures 1E–G**).

To explore individual DEG correlations with OS, we performed Kaplan-Meier survival curve analysis. In total, 421 DEGs out of 1,574 significantly predicted OS in the log-rank test (p < 0.05, selected genes are shown in **Supplementary Figure S3**).

### 3.3 Protein-protein interaction (PPI) of genes of prognostic value

To better understand interactions between prognostic value DEGs, we examined protein-protein interaction (PPI) networks in STRING. The network consisted of eight modules, which included 218 nodes and 704 edges. We selected the top three important modules for further analysis (**Supplementary Figure S4**). For descriptive convenience, we termed these modules MCODE1, MCODE2, and MCODE3 modules, respectively. In MCODE1 (**Supplementary Figure S4A**), ACKR3, CXCR3, and CCR5 had higher degree values. In MCODE2 (**Supplementary Figure S4B**), several immune response key genes occupied the module center and included HLA-DRB5, HLA-DRB1, CD247, and LCK. In MCODE3 (**Supplementary Figure S4C**), IL2RG, CD8B, and CD8A were significant nodes, as they had the most connections with other module members.

### 3.4 Functional enrichment analysis of genes of prognostic value

Consistent with PPI network analysis, functional enrichment analysis of these genes also identified strong associations with immune responses. Top GO terms included extracellular region and extracellular space (**Supplementary Figure S5A**), immune response (**Supplementary Figure S5B**), and antigen binding and signalling receptor binding (**Supplementary Figure S5C**). Additionally, all pathways from Kyoto Encyclopedia of Genes and Genomes (KEGG) analysis (**Supplementary Figure S5D**) were associated with immune responses.

### 3.5 Gene Expression Omnibus (GEO) database validation

To determine if genes identified by TCGA had prognostic significance in other BC patients, we downloaded and analyzed gene expression data from 435 BC patients (GSE42568, GSE88770, GSE48390, and GSE162228) from the GEO database. Interestingly, 15 genes were significantly and prognostically related to the validation set (**Supplementary Figure S6**, p < 0.05); NPY1R, CELSR2, STC2, SCUBE2, GIMAP2, HLA-DPB1, TFF1, CXCL14, KLRB1, BIRC3, IL18, PSMB8, CD1C, TNFAIP8, and IRF1.

### 3.6 Constructing a prognostic risk model based on TME-related genes

**Figure 2:**
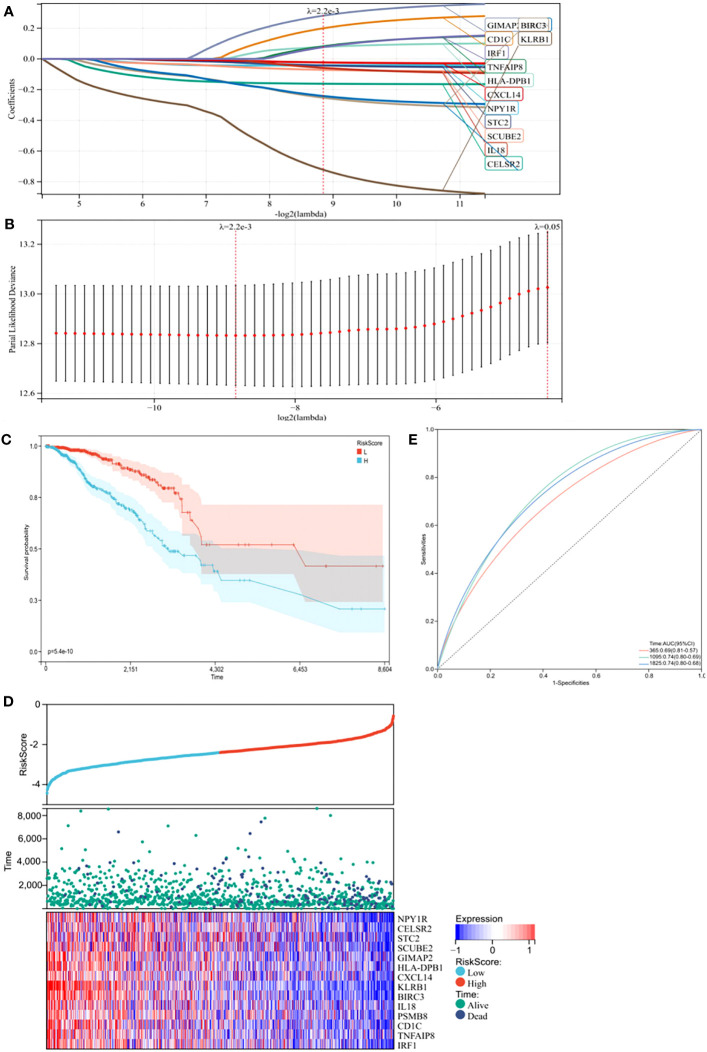
Construction of a prognostic model in the training cohort. **(A)** The Least absolute shrinkage and selection operator (LASSO) Cox regression analysis identified 14 genes most correlated with prognostics. **(B)** The optimal values of the penalty parameter were determined by 10-round cross-validation. **(C)** Patients in the high-risk group (blue) exhibited worse overall survival (OS) than those in the low-risk group (red). **(D)** Distribution of risk scores, survival profiles, and heat maps showing characteristic expressions of the low and high risky groups. **(E)** Time-dependent receiver-operating characteristic (ROC) curve.

Subsequently, we performed least absolute shrinkage and selection operator (LASSO) Cox regression analysis to select highly relevant genes from these 15 genes. Finally, 14 were identified as related to the TME in BC, and optimal values of the penalty parameter were determined by 10-fold cross-validation (**Figures 2A, B**). We then constructed a prognostic model based on these genes, with the risk score of each sample from the training cohort calculated according to this model. Based on median risk score, BC samples from the training cohort were divided into high- and low-risk groups. To assess the OS in these groups, Kaplan-Meier curves were generated and showed that OS in the high-risk group was worse than that in the low-risk group, indicating the validity of the risk score prediction (**Figure 2C**, p < 0.0001). Additionally, the expression of the TME-related genes, survival status, and survival time distribution for patients according to risk scores are shown in **Figure 2D**. In terms of model diagnosis, the AUC of the time-dependent receiver operating characteristic (ROC) curves were 0.69 for 1-year survival, 0.74 for 3-year survival, and 0.74 for 5-year survival, respectively, suggesting acceptable stability of the risk model (**Figure 2E**). In addition, to explore if BC subtypes affect survival, we grouped patients according to subtypes and subsequently performed survival analyses. Clearly, no differences in survival due to subtypes were observed, suggesting that the BC subtype did not affect survival (**Supplementary Figure S7**, p = 0.26). Together, our risk model, constructed from TME-related genes, appeared to accurately predict prognosis in BC patients.

**Figure 3:**
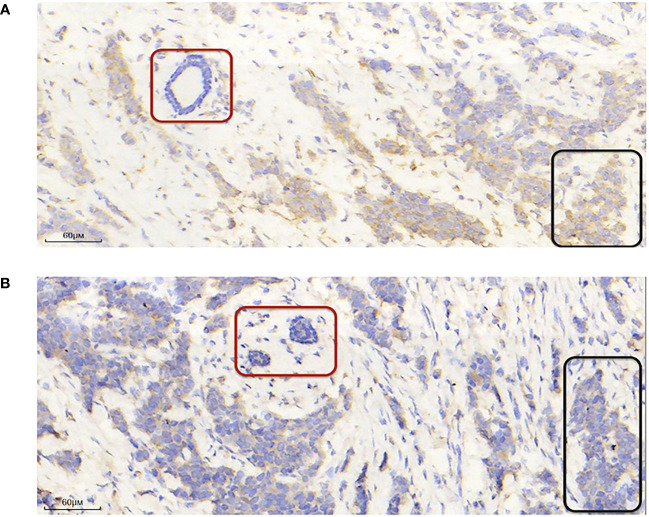
PSMB8 and BIRC3 expression. **(A)** Representative immunohistochemical image showing high and low PSMB8 expression. **(B)** Representative immunohistochemical image showing high and low BIRC3 expression. The red area indicates paracarcinoma epithelial cells and the black area indicates breast cancer epithelial cells.

Next, to identify hub genes, we identified interactions between genes in the TME model by constructing a PPI network in STRING. The network included 13 nodes and six edges. PSMB8 and BIRC3 had the maximum neighboring genes and were identified as hub genes. The Kaplan-Meier analysis showed both were the prognostic indicators, and its high expression favored the prognosis (**Supplementary Figure S2**, p < 0.05). To verify this phenomenon still exists in the human body, we used immunohistochemistry to compare hub protein expression and identified high PSMB8 and BIRC3 expression trends in BC epithelial cells when compared with paracancerous cells (**Figure 3**).

### 3.7 The risk model is an independent BC prognosis indicator

**Figure 4:**
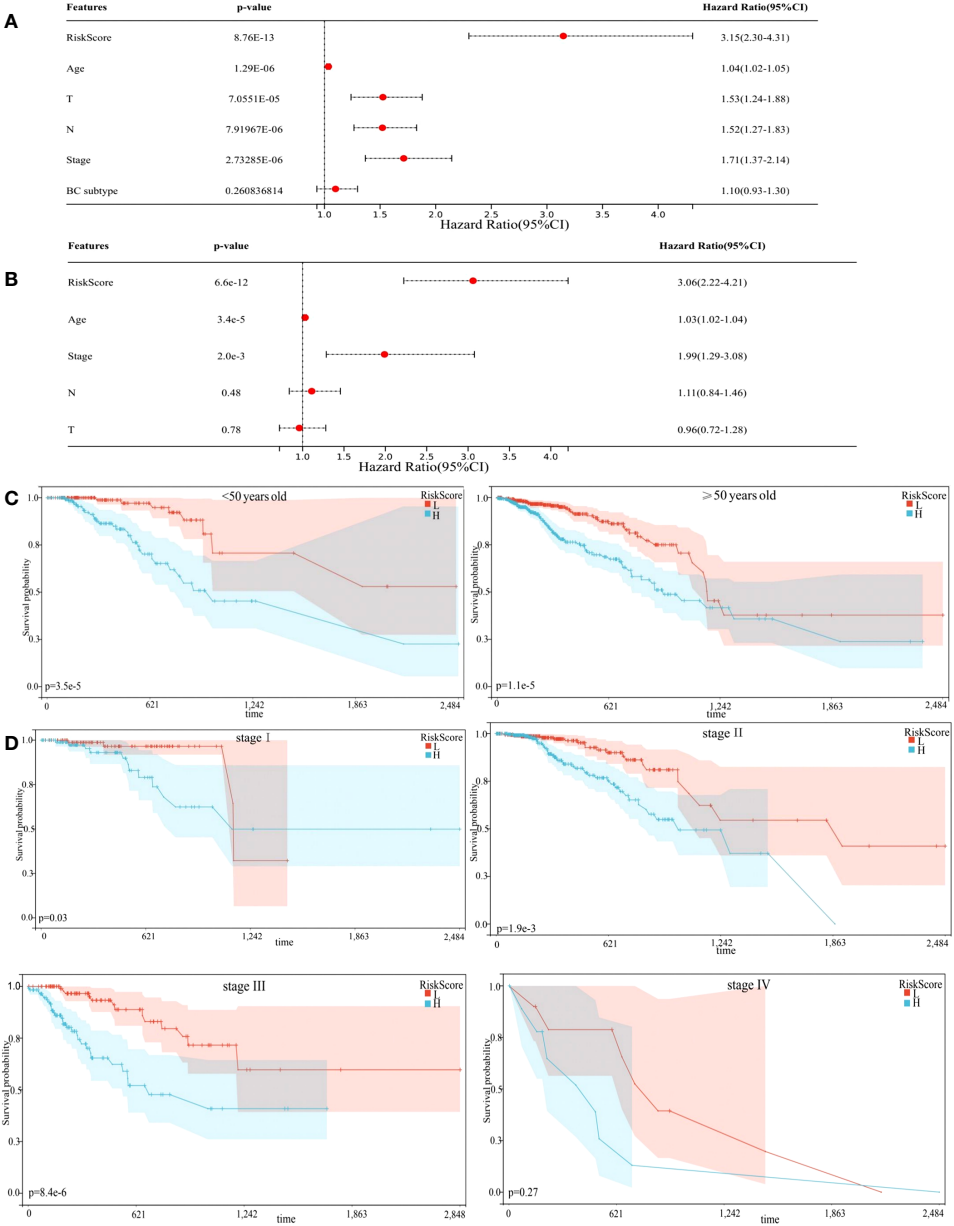
Prognostic model independence. **(A)** Results of Univariate Cox regression analysis. **(B)** Results of multivariable Cox regression analysis. **(C, D)** Subgroup analyses suggesting the independence of the prognostic model regarding age, and TNM stage.

Univariate Cox regression analysis showed that risk score could predict the prognosis of BC patients (**Figure 4A**, p < 0.0001). In the multivariable Cox regression analysis, risk score remained statistically significant (**Figure 4B**, p < 0.0001), indicating our risk model was an independent prognostic factor for BC. Additionally, BC patients in the training cohort were regrouped into subgroups based on age (< 50 and ≥ 50 years old), and TNM stage (stage I, stage II, stage III, and stage IV). Regardless of subgroups, low-risk group patients still showed significantly longer survival (**Figures 4C, D**, p < 0.05), which indicated excellent risk model independence.

### 3.8 Establishing a nomogram

**Figure 5:**
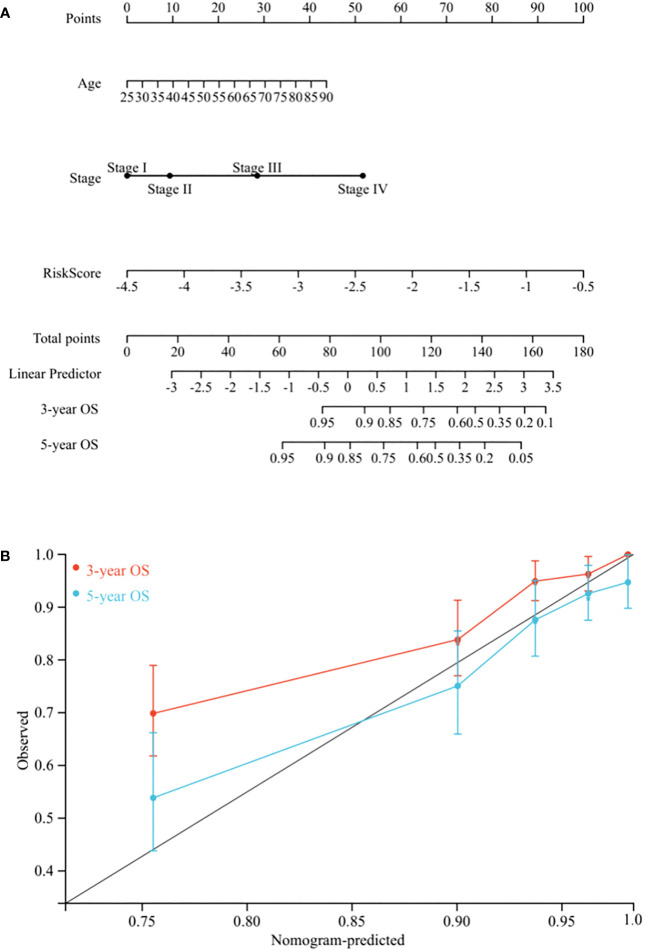
Nomogram construction. **(A)** Nomogram predicting 3-, and 5-year OS for BC patients in the training cohort based on risk score and other clinicopathological parameters (age and TNM stage). **(B)** The calibration curves of nomograms between predicted and observed 3- and 5-year OS in the training cohort. The gray line of 45° represents the perfect prediction of the nomogram.

To create a quantitative method to predict OS, we integrated the risk score and independent clinicopathological prognostic factors, including age and TNM stage, to construct a nomogram (**Figure 5A**).

To evaluate its prognostic value, we compared the concordance index (C-index) of the nomogram with TNM stage, and as shown in **Table 1**, the nomogram improved the prediction accuracy for BC. We compared predicted 3- and 5-year survival probabilities with actual probabilities and observed the calibration curve showed good concordance between these probabilities, thereby reflecting high nomogram accuracy and dependability (**Figure 5B**). Taken together, the nomogram, which integrated risk score, showed good performance and applicability, and has potential as a clinical tool to predict prognosis in BC patients.

**Table 1:** The concordance indexes of tumor-node-metastasis (TNM) stage and nomogram system.

	C-index	95% Confidence Interval
Nomogram	0.800	0.76-0.84
TNM stage	0.763	0.72-0.81

### 3.9 Correlations between the risk model and clinicopathological features

**Figure 6:**
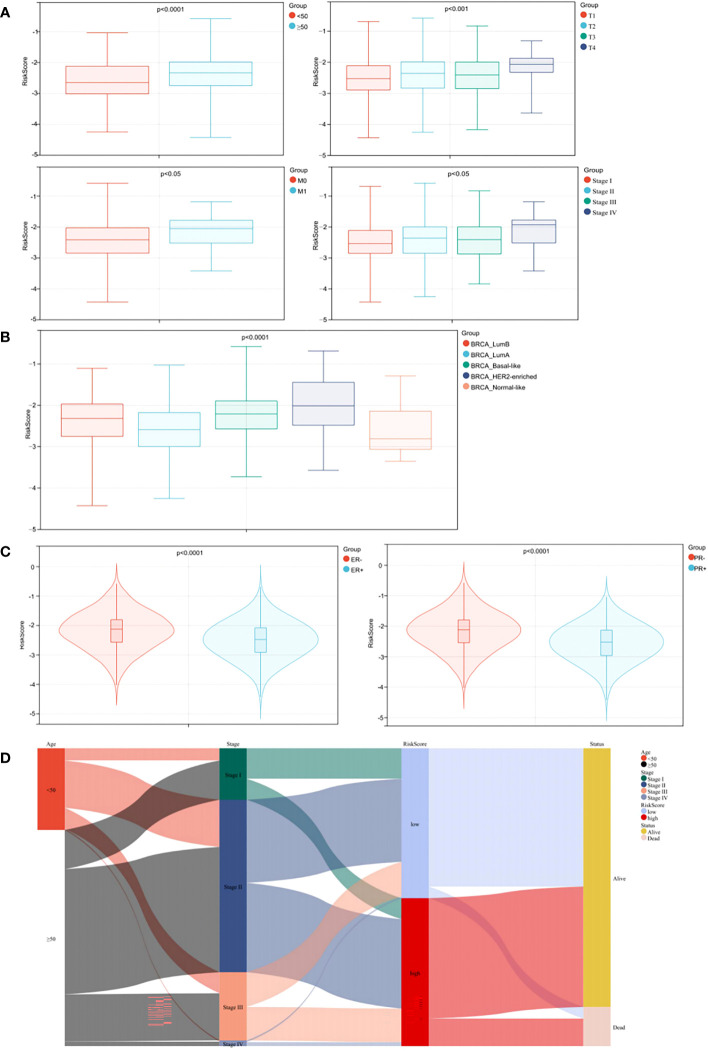
Stratified analysis of clinical characteristics for risk score in the prognostic model. **(A)** Correlation analysis of the risk score and the clinical characteristics. **(B)** Correlation analysis of the risk score and the BC subtypes. **(C)** Correlation analyses of the risk score and the status of PR/ER. **(D)** Alluvial diagram.

Relationships between prognostic risk score and clinical characteristics were further investigated in the training cohort. Age, T category, M category and TNM stage were significantly related to risk score, whereas gender and N category were not (**Figure 6A**, p < 0.05). As observed **Figure 6B**, patients with HER2-enriched had the highest risk score, followed by Basal-like, Luminal B, and Luminal A subtypes, while Normal-like patients had the lowest scores (p < 0.0001). Association analysis with hormone receptor status showed that patients with PR+/ER+ had lower risk score when compared with PR-/ER- patients, and ER+ patients had lower risk score when compared with ER- patients (**Figure 6C**, p < 0.0001).

To better visualize the clinicopathological features in individual patients and assess correlations with survival, we used an alluvial diagram which showed that risk categories in the prediction model accurately predicted patient survival (**Figure 6D**).

### 3.10 Correlation between the risk model and immune infiltration

**Figure 7:**
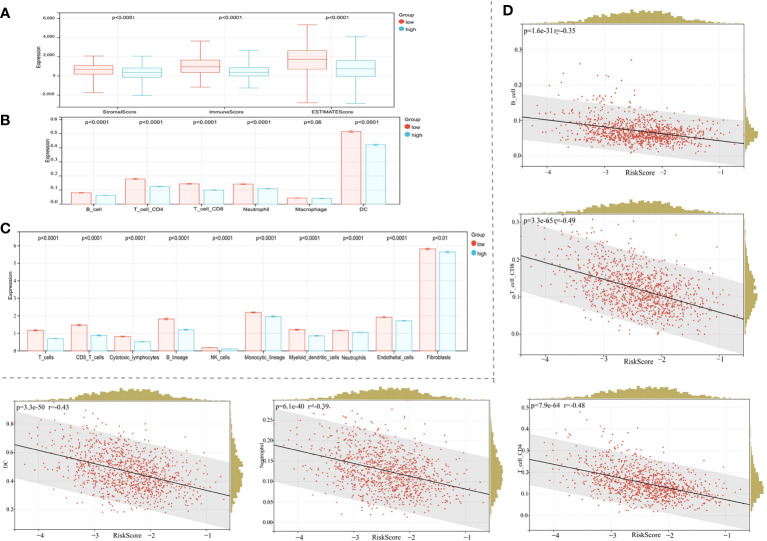
Correlation between the risk model and the immune microenvironment. **(A)** The ESTIMATE algorithm. **(B)** The TIMER algorithm. **(C)** The MCPcounter algorithm. **(D)** Correlations between the risk score and the infiltration of immune cell subtypes.

Association between the risk model and immune cell infiltration was assessed using several immune infiltration approaches. ESTIMATE algorithm data showed that immune, stromal, and ESTIMATE scores in the high-risk BC patient group were lower when compared with BC patients in the low-risk group (**Figure 7A**, p < 0.0001). The TIMER algorithm showed that B cell, neutrophil, CD4 T cell, dendritic cell (DC), and CD8 T cell abundance, but not macrophage, was statistically higher in the low-risk group when compared with the high-risk group (**Figure 7B**, p < 0.0001). Moreover, MCPcounter algorithm results showed that T cells, CD8 T cells, cytotoxic lymphocytes, B lineage cells, natural killer (NK) cells, monocytic lineage cells, myeloid DCs, neutrophils, endothelial cells, and fibroblasts were highly infiltrated in the low-risk group (**Figure 7C**, p < 0.01). Thus, our risk model correlated well with different immune microenvironment components.

Given the significant correlation of our risk model with the BC immune microenvironment, we next examined relationships between the risk model and immune cell subtype infiltration using Pearson’s algorithm. As shown in **Figure 7D**, correlation values for B cells, CD4 T cells, CD8 T cells, DCs, and neutrophils with risk scores were −0.35, −0.48, −0.49, −0.43, and −0.39, respectively. As expected, immune cell infiltration levels were significantly and positively correlated with prognosis (**Figure 7D**, p < 0.0001).

### 3.11 Practical analysis of the risk model

**Figure 8:**
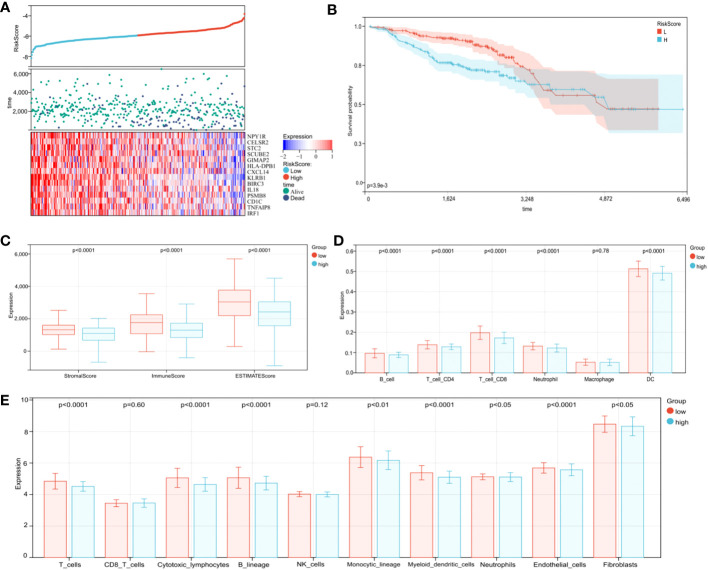
Validation of the prognostic risk model in the validation cohort. **(A)** Distribution of risk scores, survival profiles, and heat maps showing characteristic expressions of the low- and high-risk groups. **(B)** Patients in the high-risk group (blue) exhibited worse overall survival (OS) than those in the low-risk group (red). **(C)** The ESTIMATE algorithm. **(D)** The TIMER algorithm. **(E)** The MCPcounter algorithm.

To further confirm model practicability and reliability, it was verified using a validation cohort. Risk scores, survival status, and gene expression are shown in **Figure 8A**. As expected, significant differences in OS were identified between groups, with longer OS in the low-risk group (**Figure 8B**, p < 0.0001). Furthermore, relationships between risk score and the BC immune microenvironment were confirmed in the validation cohort. From ESTIMATE, TIMER, and MCPcounter analysis, the low-risk group was significantly associated with high immune cell infiltration levels From ESTIMATE analysis, the low-risk group was significantly associated with high stromal, immune, and ESTIMATE scores (**Figure 8C**, p < 0.0001). In TIMER analysis, the abundance of the five aforementioned immune cell types, except macrophages, was statistically different between groups (**Figure 8D**, p < 0.0001), and immune cell abundance (all types) was significantly higher in the low-risk group than the high-risk group. The MCPcounter algorithm showed that T cells, cytotoxic lymphocytes, B lineage, monocytic lineage cells, myeloid DCs, endothelial cells, neutrophils, and fibroblasts were in a high infiltration state in the low-risk group (**Figure 8E**, p < 0.05). Therefore, our TME-related gene risk model was associated with BC prognosis and the immune microenvironment.

### 3.12 The risk model predicts chemotherapy efficacy

**Figure 9:**
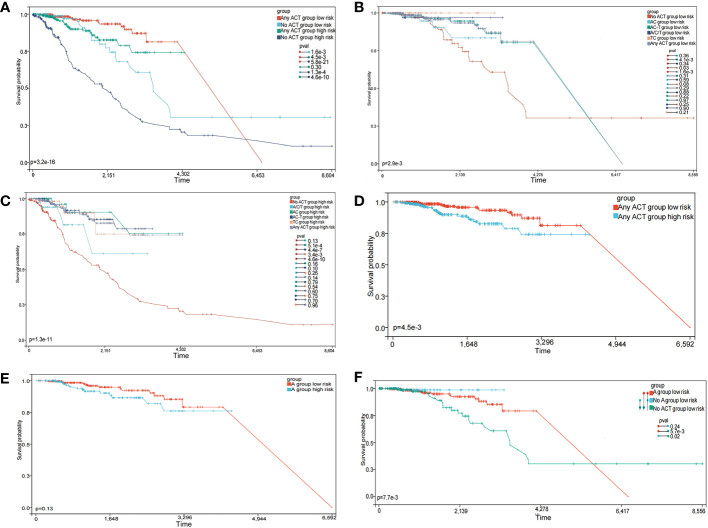
The prognostic model predicts chemotherapy efficacy. **(A)** Subgroup analysis of adjuvant chemotherapy (ACT) benefit for overall survival (OS) of low-and high-risk patients in the TCGA database. **(B)** OS analysis in patients with different chemotherapy regimens in the low-risk group. **(C)** OS analysis in patients with different chemotherapy regimens in the high-risk group. **(D)** OS analysis of treated patients in high- and low-risk groups. **(E)** OS analysis of patients receiving the anthracycline **(A)** regimens in high- and low-risk groups. **(F)** OS analysis of patients receiving A, no-A, and no treatment in the low-risk group.

As neoadjuvant and adjuvant chemotherapies are reportedly related to immune infiltration (37), we evaluated if chemotherapy influenced BC prognosis. According to the NCCN Guidelines in Oncology, anthracycline + cyclophosphamide (AC), AC followed by taxane (AC-T), and taxane + cyclophosphamide (TC) are major chemotherapy regimens. The OS advantage was observed in the low-risk group, regardless of whether they received chemotherapy or not. And whether in high-risk group or low-risk group, patients who received chemotherapy had a better prognosis (**Figure 9A**, p < 0.0001). In the low-risk group, the OS advantage was evident in patients who received TC and AC-T chemotherapy regimens when compared with those who received no chemotherapy (**Figure 9B**, p < 0.05). In contrast, the chemotherapy benefits in the high-risk group were observed for AC, TC, and AC-T chemotherapy regimens (**Figure 9C**, p < 0.05). More importantly, subgroup interaction evaluations suggested that better chemotherapy outcomes were achieved in low-risk patients regardless of the chemotherapy regimen (**Figure 9D**, p < 0.05).

We also explored if the A-regimen was an indispensable chemotherapy agent in the low-risk group. As shown in **Figure 9E**, no significant differences in prognosis outcomes for patients treated with the A-regimen were identified, regardless of low- or high-risk (p > 0.05). Further subgroup analysis showed no significant differences in prognosis outcomes in low-risk patients who received the A-regimen when compared with those who did not (**Figure 9F**, p > 0.05). These observations suggested that the low-risk group selected by this prediction model has the opportunity to exempt the A-containing adjuvant chemotherapy regimen.

### 3.13 The risk model predicts gene expression in immune responses, immune checkpoints, inflammation, and epithelial-mesenchymal transition

**Figure 10:**
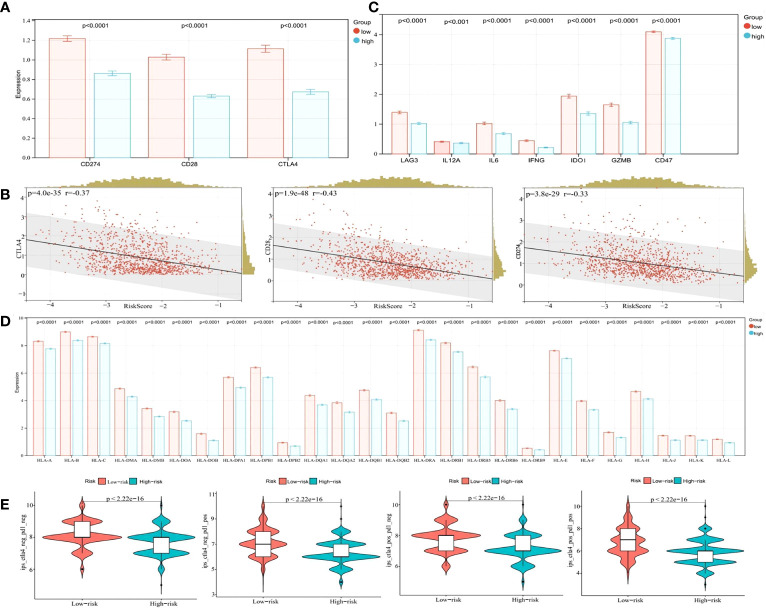
Bioinformatics analysis of the characteristics and signaling pathways among patients in different risk groups. **(A)** CD274, CD28, and CTLA4 mRNA expression between the low- and high-risk groups in the cohort from TCGA. **(B)** Correlation between the risk score and CD274, CD28, and CTLA4 mRNA expression. **(C)** LAG3, IL12A, IL12B, IL6, IFNG, IDO1, GZMB, and CD47 mRNA expression between the low- and high-risk groups in the cohort from TCGA. **(D)** The HLA family mRNA expression between the low- and high-risk groups in the cohort from TCGA. **(E)** Correlation of the risk score and the IPS.

Immune checkpoint blockade with immunotherapies, including CTLA-4, CD28, and CD274 are promising treatment approaches for several malignancies (38). However, the bottleneck problem of immune checkpoint inhibitors (ICI) in the treatment of eBC is the lack of precise biomarkers identifying populations who may benefit from these therapeutics. In our study, we determined the expression levels of several key immune checkpoint regulators and inflammatory mediators to provide reference biomarker candidates for precision immunotherapy in early drug-resistant patients. As presented in **Figure 10A**, CD274, CD28, and CTLA-4 expression levels were significantly higher in the low-risk group (p < 0.0001). The Pearson algorithm was used to analyze correlations between immune checkpoints and our risk model. Correlation values of CTLA-4, CD28, CD274 and risk score were -0.37, -0.43 and -0.33, respectively (**Figure 10B**, p < 0.0001). Additionally, other immunomodulators or inflammatory mediators were increased in the low-risk group (**Figure 10C**, p < 0.0001). A previous study reported that HLA affected ICI efficacy (39), therefore we analyzed correlations between HLA family expression and our model, and showed this expression was significantly higher in the low-risk group when compared with the high-risk group (**Figure 10D**, p < 0.0001). We next explored ICI therapy responses, represented by the CTLA-4/PD1 inhibitors, by using the immunophenotype score (IPS), and showed that the IPS was slightly higher than that of the low-risk group in the patients treated with CTLA-4 and PD1 inhibitors (**Figure 10E**, p < 0.05). Overall, these results suggested that our model predicted the immunotherapy benefits for patients and may be a more effective biomarker to predict the efficacy of immunotherapy.

We further analyzed DEGs between low- and high-risk groups in TCGA. In total, 396 DEGs (7 upregulated and 389 downregulated genes, FDR p-value < 0.05) were identified in the high-risk group when compared with the low-risk group. Of these, SLC7A5, PRAME, CRABP1, CBX2, CA9, CALML5, and CD24 were significantly overexpressed in the high-risk group (**Supplementary Figures S8A, B**, FDR p-value < 0.05, fold change > 1.5). Furthermore, KEGG analysis showed that genes in the high-risk group were mainly involved in environmental information processing, human diseases, and organismal systems (**Supplementary Figure S8C**). From GO enrichment analysis, these genes in the high-risk group were mainly involved in extracellular matrix, vesicle, immune response, and antigen binding (**Supplementary Figures S8D–F**).

### 3.14 Risk model correlation with tumor mutation burden (TMB)

**Figure 11:**
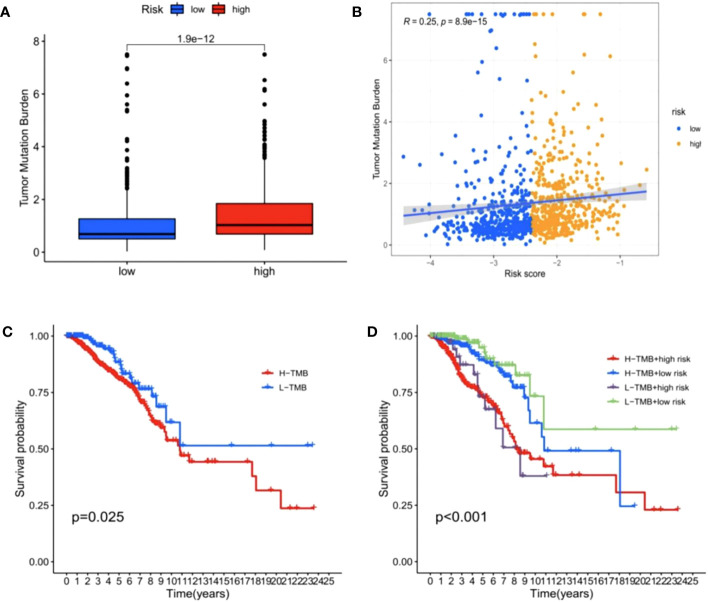
Correlations between risk score and tumor mutation burden (TMB). **(A)** The TMB was higher in the high-risk group than in the low-risk group. **(B)** The scatterplots depicted the positive correlation between the risk score and TMB. **(C)** Kaplan–Meier curves of overall survival (OS) in different TMB subgroups. **(D)** Kaplan–Meier curves of overall survival (OS) stratified by both TMB and the risk score.

As shown in **Figure 11A**, BC patients in the high-risk group had a higher TMB than those in the low-risk group (p < 0.05). As suggested from previous studies, a high TMB leads to a poor prognosis in many cancers (40), consistent with our data. In correlation analysis between risk score and TMB, we identified a significant positive correlation (**Figure 11B**, p < 0.05). Further survival analysis indicated that the low-TMB group showed a significant survival benefit (**Figure 11C**, p < 0.05). Given the synergistic effect of TMB and the risk score, their effect on prognostic stratification was evaluated. As indicated from the results, TMB status did not interference the predictive ability of the risk score. Survival difference of the risk score subtypes was significant in both high- and low-TMB groups, and the subgroup with low risk-score and low TMB showed a better survival benefit, while the high-risk score and high TMB subgroup had a lower survival probability (**Figure 11D**, p < 0.001). Combined, risk score may act as a prognostic BC indicator, which is independent of TMB and can effectively predict TMB and treatment sensitivity.

### 3.15 Relationships among hub genes expression levels, tumor-infiltrating immune cells, immune molecules, and sensitivity to BC-targeting and chemotherapeutic drugs

**Figure 12:**
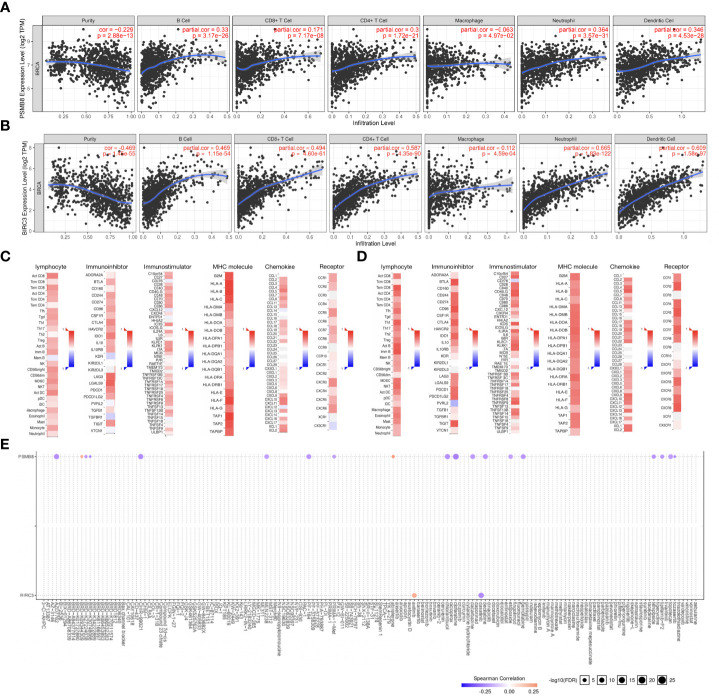
Relationships between hub gene expression and tumor-infiltrating immune cells, immune molecules, and sensitivity to BC-targeting and chemotherapeutic drugs. **(A)** Upregulation of PSMB8 expression is associated with increased infiltration of B cells, CD8+ T cells, macrophages, neutrophils, dendritic cells (DCs), and other infiltrating lymphocytes. **(B)** Upregulation of BIRC3 expression is associated with increased infiltration of B cells, CD8+ T cells, macrophages, neutrophils, dendritic cells (DCs), and other infiltrating lymphocytes. **(C)** The correlation between PSMB8 expression and lymphocytes, immunostimulatory molecules, immunosuppressive molecules, MHC molecule, chemokines, and chemokine receptors in BC. **(D)** The correlation between BIRC3 expression and lymphocytes, immunostimulatory molecules, immunosuppressive molecules, MHC molecule, chemokines, and chemokine receptors in BC. **(E)** The expression levels of PSMB8and BIRC3 are correlated with sensitivity to many BC-targeting and chemotherapeutic drugs.

We used the TIMER database to explore the relationships between hub genes expression (PSMB8 and BIRC3) and the level of infiltrating lymphocytes. Upregulated PSMB8/BIRC3 expression was associated with increased B cell, CD8+ T cell, macrophage, neutrophil, DC, and other infiltrating lymphocyte infiltration (**Figures 12A, B**, p < 0.05). Next, using the TISIDB database, we found that upregulated PSMB8/BIRC3 (**Figures 12C, D**) expression was associated with increased expression of immunostimulatory molecules, immunosuppressive molecules, MHC molecules, chemokines, and chemokine receptors, which provides important information for predicting potential therapeutic targets. Finally, we used GSCA Lite online tool to analyze the relationship between the expression of the hub genes and sensitivity to current immune or targeted therapies for BC (**Figure 12E**). PSMB8 expression levels were negatively correlated with sensitivity to many BC-targeting or chemotherapeutic drugs, including clofarabine and gemcitabine, and were positively correlated with abiraterone. BIRC3 expression levels were positively correlated with axitinib sensitivity and negatively correlated with dasatinib sensitivity. Thus, hub genes could function as new targets for predicting drug sensitivity and developing multi-targeted combined therapy for BC.

## 4 Discussion

We developed a 14-TME-related gene prognostic model based on statistical associations between eBC prognosis and drug resistance. (1) Our model exhibited strong predictive prognosis power in BC patients; (2) Enrichment analyses showed that immune-related pathways mediated the role of TME-related genes in BC; (3) we constructed a nomogram system, which was shown when compared with simple clinicopathological features, nomogram-integrated risk score had high prediction accuracy and applicability; (4) Our model provided predictive power for eBC patients to select the best treatments possible and avoid unnecessary chemotherapy agents; and (5) We found 2 novel therapeutic target genes, which provides a new direction for the development of BC precision medicine.

With the wide application of high-throughput technology and the continuous maturity of data sharing mechanism, unprecedented large-scale multi-omics cancer data have been accumulated in the international public databases, and cancer research has entered the era of “big data”. The focus of precision genomic medicine is to identify accurate specific survival prognostic factors from large medical datasets with clinical outcomes (41). Therefore, in recent years, some studies have aimed to explore microenvironment-related prognostic factors using bioinformatics analysis. However, the use of genomics, transcriptomic, and proteomic analysis of clinical tumor tissue is affected by the proportion of tumor cells present, and the method of evaluating the nontumor part of tumor samples (ESTIMATE) can provide an important context for genomic data analysis, a huge improvement in other capacity-limited methods (42). Additionally, many studies have not comprehensively explored the role of the genes related to stromal cells and immune cells in the BC TME and focused only on immune cell-related genes. In this study, we investigated infiltrating immune and stromal cell levels in tumor tissue in the ESTIMATE algorithm, and provided new perspectives for the comprehensive understanding on tumor-related normal cells in tumor tissue.

In our study, we used the ESTIMATE algorithm to assess the levels of infiltrating immune and stromal cell levels in tumor tissues. And we showed that the Basal-like subtype had a high immune score, consistent with previous findings showing that high levels of TILs were common in both the Basal-like type and the HER2-enriched types (43). The effect of tumor-infiltrating immune cells on the biological and clinical course of BC is well established in previous research (44). In accordance with the previous studies, we observed that BC patients with higher immune scores had the better prognosis, while no significant association of stromal scores with prognosis was observed. For another, LASSO regression was applied to construct risk models for 14 key TME prognostic genes, as used in previous studies (45, 46). The prognostic value of our risk model was also confirmed in the training and validation sets. The OS curves of the high-risk scoring group and low-risk scoring group were obviously separated, and patients with low-risk scores comprised a clear survival advantage, which vindicated our study design. The fly in the ointment was that we observed similar survival rates with the high- and low-risk groups in the validation set at late time points. Studies have shown that the survival curves crossing happens, when a relative few subject still being followed at late time points. When the sample reduce, there will also be a lot of uncertainty in the true position of the survival curves (47). Consistent with this, our data and results shown that the samples in the later stage of this survival curve have been reduced a lot compared to those at the start (**Supplementary Figure S9**). In addition, insufficient samples, differences in patient treatment regimens, and age deviation may also contribute to this phenomenon. Furthermore, model diagnosis using ROC analysis indicated that our risk model was a reliable indicator for predicting prognosis. Subgroup analysis further showed that risk score remained independent prognostic factor even when patients were regrouped based on clinical parameters. Finally, a nomogram, which may be used in clinical practice, was constructed and a calibration curve used to explore the predictive efficacy of our model for survival. Overall, our risk model of TME-related genes may be a mature reference for predicting prognosis in patients with BC that is feasible in clinical practice.

In this study, we selected 14 TME-related genes, including BIRC3, CELSR2, CXCL14, IL18, KLRB1, NPY1R, PSMB8, SCUBE2, STC2, CD1C, HLA-DPB1, GIMAP2, IRF1 and TNFAIP8, all of which were implicated in tumor progression and prognosis outcomes. BIRC3 is a member of the apoptosis inhibitor (IAP) family, with pro-survival and antiapoptotic effects in cancer cells (48). BIRC3 is associated with treatment resistance in BC; IL-1 upregulates BIRC3 and generates doxorubicin resistance in BC cells (49), thus BIRC3 appears to have important roles in the TME. PSMB8 is the catalytic subunit of the immunoproteasome and is implicated in glioblastoma, mucinous ovarian cancer, cutaneous squamous cell carcinoma, papillary thyroid carcinoma, and prostate cancer development and progression (50–52), consistent with our findings showing that PSMB8 was associated with high immune infiltration and was a predictive protective gene. CELSR2 is part of the cadherin superfamily and was associated with poor prognosis (53). However, we confirmed CELSR2 was a protective gene and involved in changing the TME. These contradictory results highlight the need for more experimental studies on CELSR2. Furthermore, we found the first prognostic value of CD1C and GIMAP2 genes, which may provide new directions for further BC research.

In recent years, tumor immunity has attracted considerable research interest, while prognostic features related to the TME have great applications in identifying novel biomarkers. As described, BC growth and invasiveness are influenced by different cells in the TME. Many studies have reported that the degree of immune infiltration in the TME correlates with BC prognosis (30, 54). GO and KEGG analysis indicated that the DEGs between the high-risk and low-risk groups were mainly enriched in immune-related pathways. Specifically, ESTIMATE, TIMER and MCPCounter analysis showed that patients in the low-risk group had a relatively high immune infiltration status. When combined with the patient survival results, we showed that a good prognosis is associated with a high immune infiltration status, consistent with previous studies (30, 54). In the TME, tumor cells interact with different immune cell types by activating the immune checkpoint pathway (55, 56). We identified several immune checkpoint genes (e.g., CTLA-4, PDL1, LAG3, and CD28) which were highly expressed in the low-risk group, suggesting these patients may benefit from immunotherapy. The genomic instability may produce an immune response phenotype that affects the immune response and immunotherapy (57). We comprehensively analyzed correlation between the TMB and risk score and identified significant positive associations. Furthermore, the stratified prognostic analysis showed that the prognostic value of the risk score in the BC was independent of the TMB. Taken together, our results provide potential therapeutic targets and provide novel clinical applications for immunotherapies.

Chemotherapy is an important adjuvant treatment for eBC but has long been regarded as an immunosuppressive treatment modality. However, recent studies reported that chemotherapy has immune modulation effects (58, 59). The induced stress and apoptosis generated by chemotherapy produces new tumor immune antigens on cell surfaces and in the TME, which stimulate antitumor immune responses (60). Our results suggested that receiving chemotherapy was better than not receiving it, regardless of the immune microenvironment in low- or high-risk groups. A-based chemotherapeutic agents are represented by topoisomerase 2 inhibitors and have pivotal roles in eBC chemotherapy. However, it also exerts dose-dependent toxic side effects such as myelosuppression, cardiotoxicity, and gastrointestinal responses (61). Based on a pooled analysis of PlanB and SUCCESS C randomized clinical trials, six TC cycles provided similar efficacy to the A-regimen in most patients with HER2-eBC, and a significantly lower incidence of overall grade 3/4 toxicity was observed (62). The randomized neoadjuvant multicenter phase II trial, WGS-ADAPT-TN, found that additional A-containing chemotherapy was not associated with a significant invasive disease-free survival advantage in pathological complete response patients (63). Therefore, A-regimen removal is the trend, but how to accurately screen the population of chemotherapy is not unclear. We observed that A-use in the high-risk group may potentially promote immune cell infiltration and enhance antitumor immune responses. Interestingly, no prognosis differences were identified between A-use in low- and high-risk groups, and even an absence of A-regimen in the low-risk group did not affect long-term survival. This suggested that the no-A chemotherapy regimen seems feasible in low-risk patients despite chemotherapy benefit. Thus, we provide clinicians with an accurate tool that provides an opportunity for patients to choose the best treatment and avoid unnecessary chemotherapy.

Our study had some limitations. Firstly, our conclusions were based on open datasets and not sequencing data. Despite this weakness, the concordance between our TME-related gene risk model and survival in TCGA and GEO cohorts identified prognostic signatures in BC, but which still need to be further validated with sufficient sample data. Secondly, our data, which originated from databases, lacked experimental validation. In future studies, we will focus on these novel molecules using *in vitro* and *in vivo* analyses.

## 5 Conclusions

We comprehensively explored the role of the TME in BC patients using statistical analyses of public database data. First, the risk model we constructed based on TME-associated genes and successfully predicted the OS in BC patients. In addition, our model was inversely associated with BC immune cell infiltration and may be used as an independent prognostic marker to predict the efficacy of immunotherapy in BC patients. Importantly, we showed that outcomes in patients receiving the A-regimen in the low-risk group were not significantly different to those receiving the no-A regimen, suggesting this patient cohort may be exempted from A-containing adjuvant chemotherapy. The hub genes (BIRC3 and PSMB8) can be used as effective biomarkers to predict BC prognosis and used as novel targets to predict drug sensitivity.

Our work provides innovative perspectives for future BC research and the development of targeted therapeutic strategies for BC patients. Further studies are required to validate the clinical prognostic value of our risk model and explore underlying mechanisms associated with eBC.

## Data availability statement

The original contributions presented in the study are included in the article/**Supplementary Material**, further inquiries can be directed to the corresponding author/s.

## Ethics statement

The studies involving humans were approved by the Ethics and Human Subject Committee of the Second Hospital of Dalian Medical University. The studies were conducted in accordance with the local legislation and institutional requirements. The participants provided their written informed consent to participate in this study.

## Author contributions

HC conceived this study and wrote the manuscript. HC, SW and YuZ executed the data collection and data analysis. YuZ and XG performed immunohistochemistry analysis. YG, NW, XW, TZ, YiZ, DC, MW, and DZ assisted revising the manuscript. JW designed the study and was the director for the fund. All authors contributed to the article and approved the submitted version.
