# Supplementary material for: A prognostic mathematical model based on tumor microenvironment-related genes expression for breast cancer patients
Source: Front Oncol. 2023 Oct 4;13:1209707. doi: 10.3389/fonc.2023.1209707 (PMC10583559; doi:10.3389/fonc.2023.1209707)
Supplement: Supplementary file 1 [file DataSheet_1.pdf]

Supplementary Material

1 Supplementary Figures

1.1 Supplementary Figures

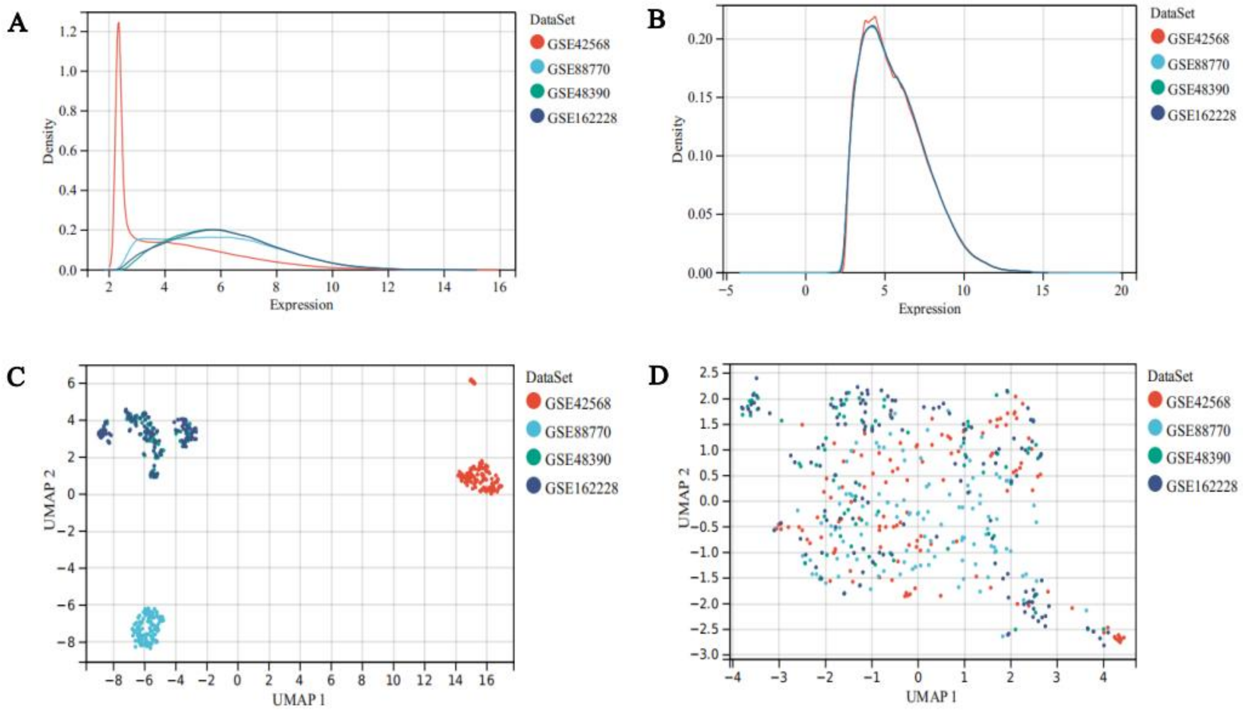

Supplementary Figure 1. Removed batch effect. **(A)** Density map before batch effect removal. **(B)** Density map after batch effect removal. **(C)** UMAP map before batch effect removal. **(D)** UMAP map after batch effect removal.

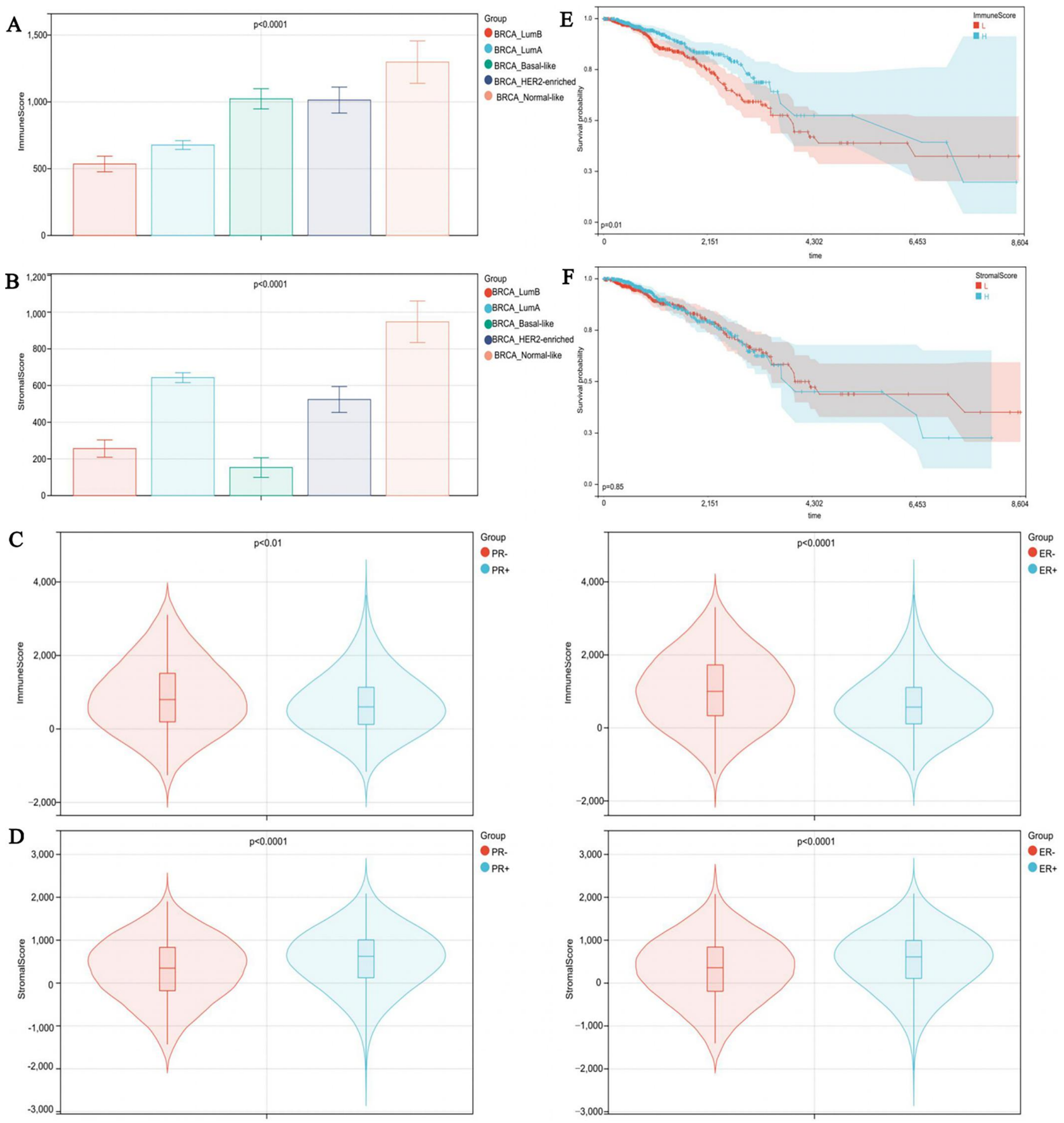

Supplementary Figure 2. Correlations between immune scores and stromal scores with BC subtypes, hormone receptor status, and overall survival (OS). **(A)** Distribution of immune scores of BC subtypes. **(B)** Distribution of stromal scores of BC subtypes. **(C)** Distribution of immune scores for PR status and ER status. **(D)** Distribution of stromal scores for PR status and ER status. **(E)** Correlations of immune scores and OS. **(F)** Correlations of stromal scores and OS.

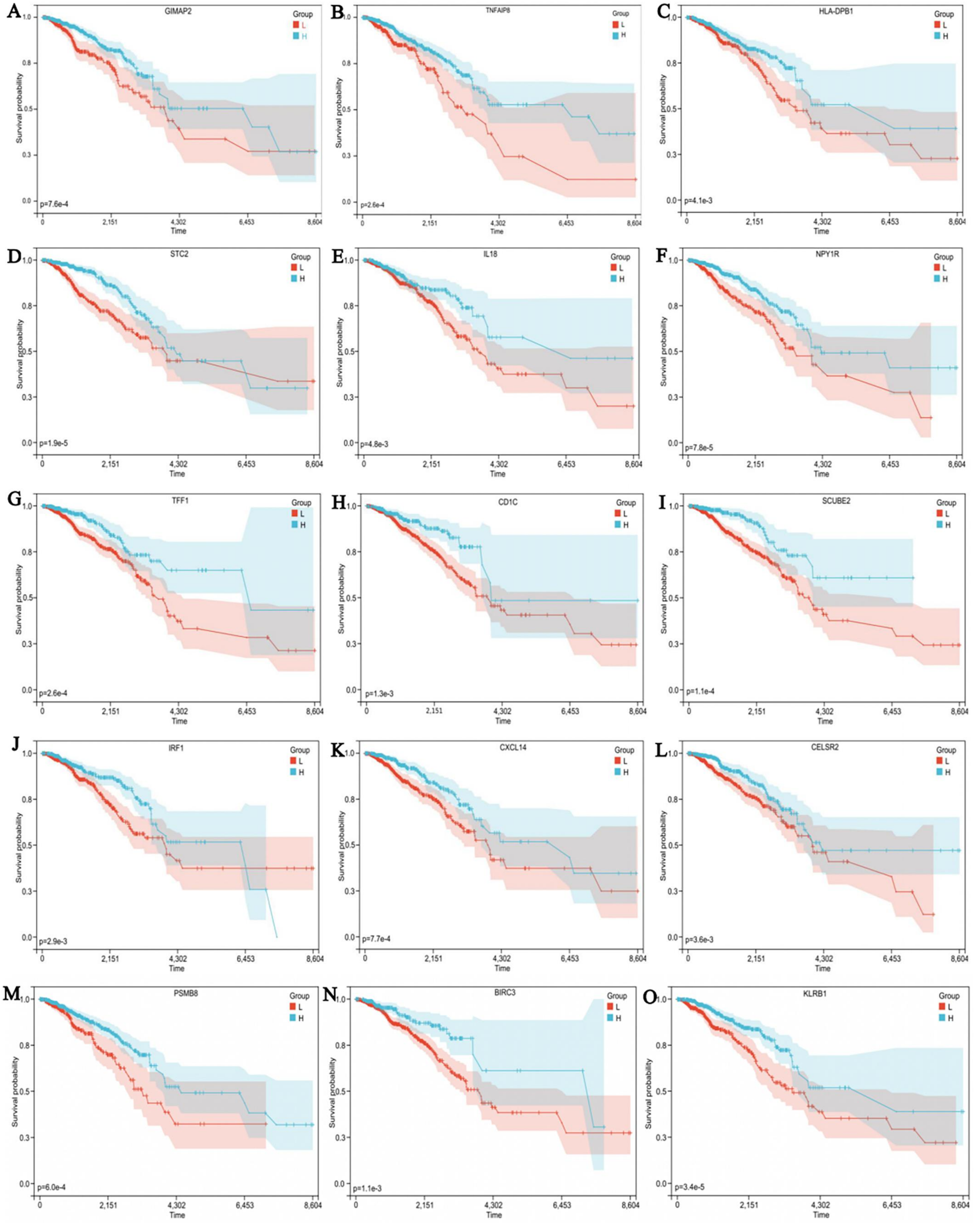

Supplementary Figure 3. Correlations between individual differentially expressed genes (DEGs) and overall survival (OS) in The Cancer Genome Atlas (TCGA).

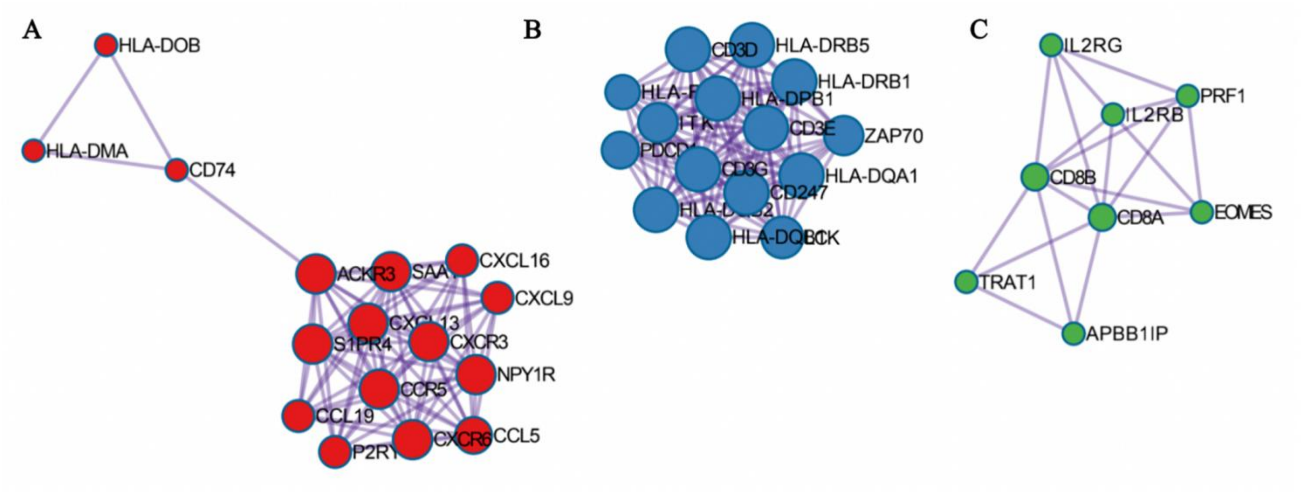

Supplementary Figure 4. Three key protein-protein interaction (PPI) networks. **(A)** MCODE1 module. **(B)** MCODE2 module. **(C)** MCODE3 module.

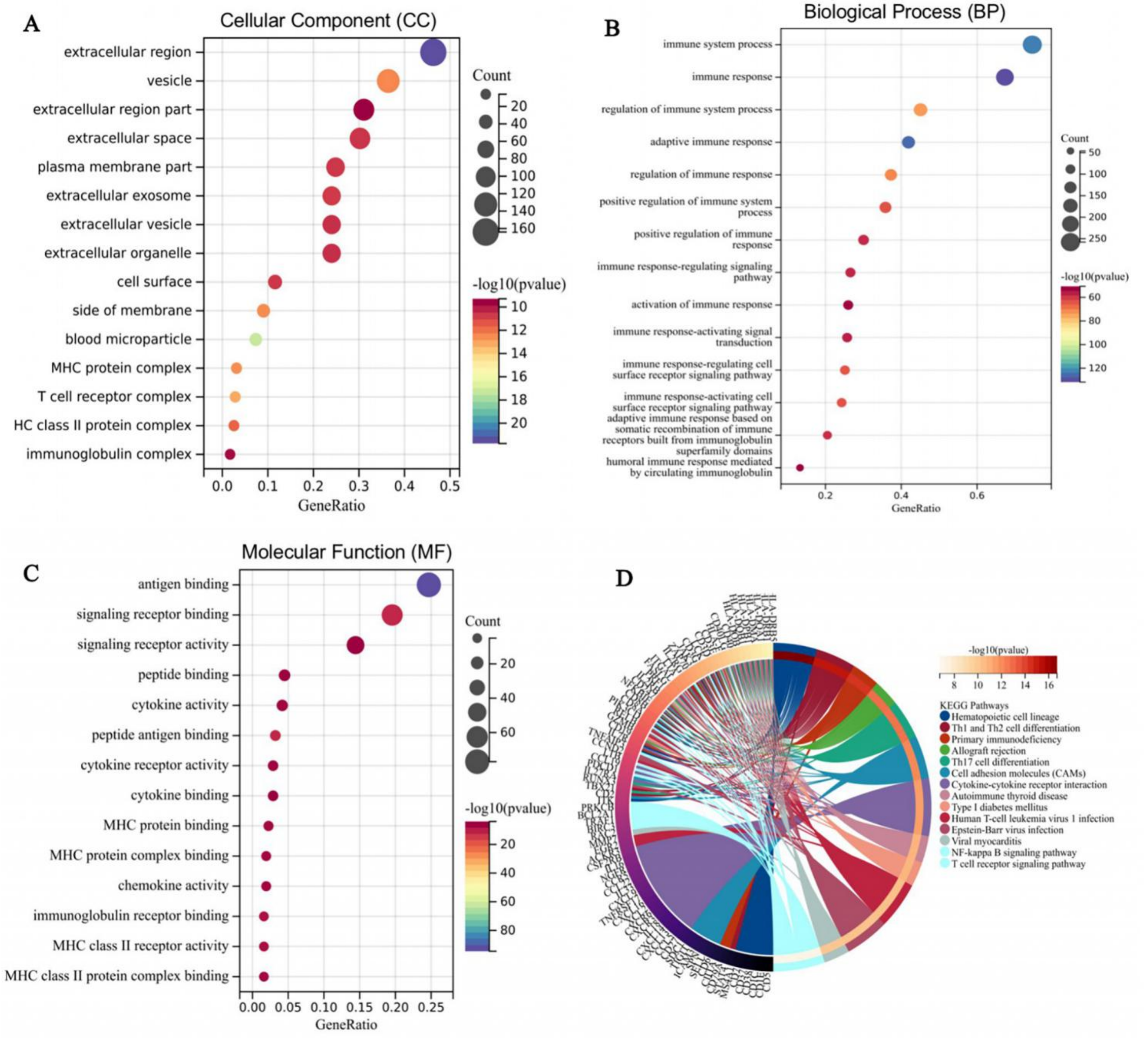

Supplementary Figure 5. Functional enrichment analysis of genes of prognostic value. **(A-C)** Gene Ontology (Go) analysis. **(D)** Kyoto Encyclopedia of Genes and Genomes (KEGG) pathway analysis.

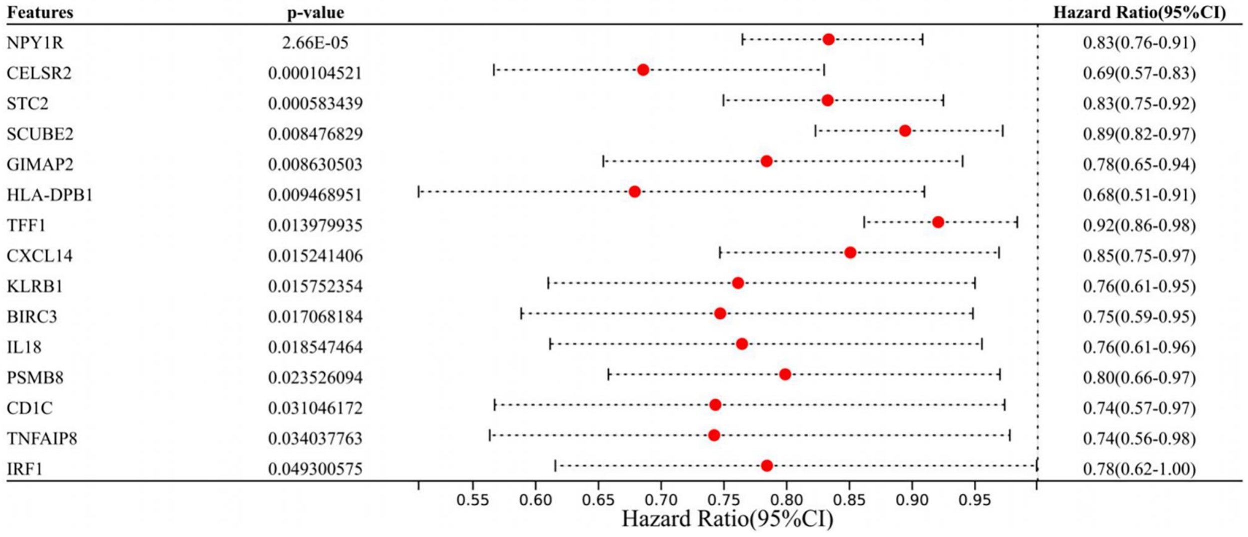

Supplementary Figure 6. Correlation validation between differentially expressed genes (DEGs) from TCGA and overall survival (OS) in the Gene Expression Omnibus (GEO) cohort.

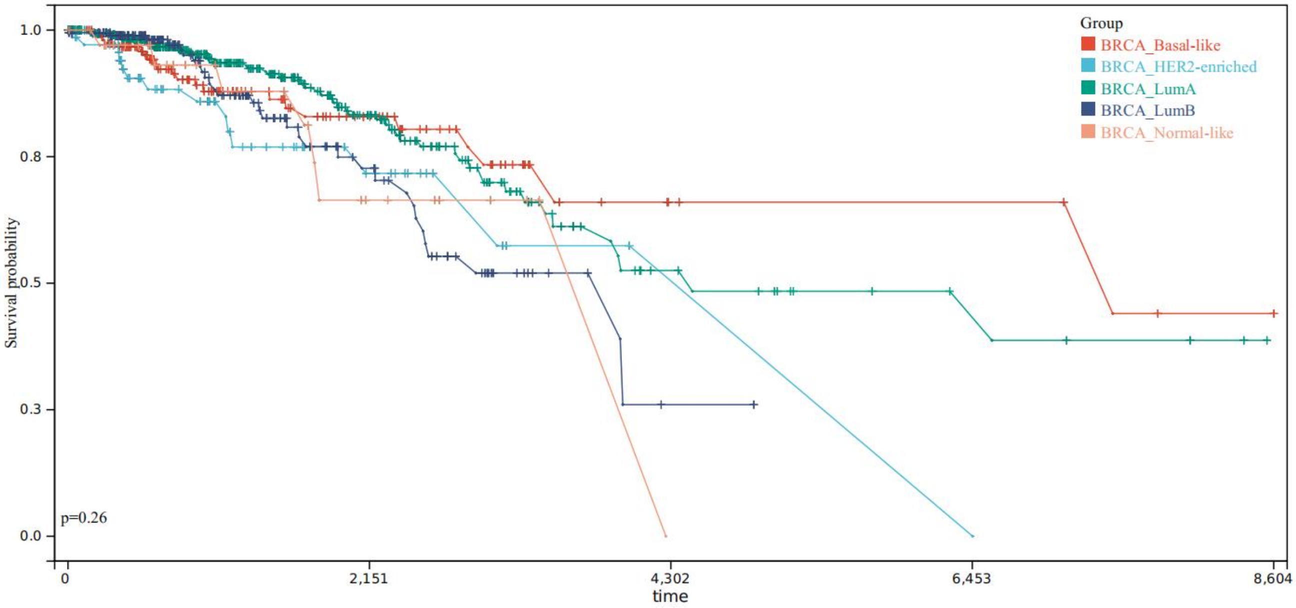

Supplementary Figure 7. Correlations of BC subtypes and OS.

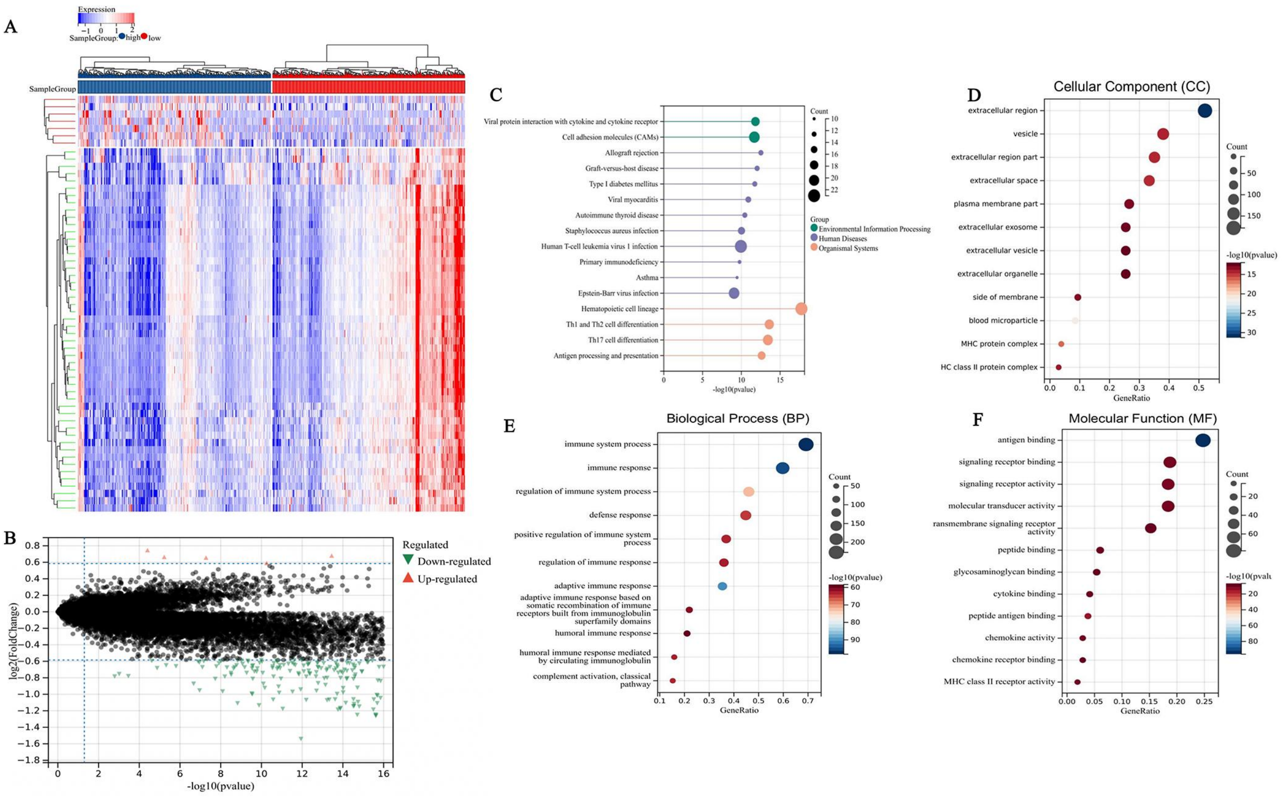

Supplementary Figure 8. Functional enrichment analysis among patients in different risk groups. **(A)** Heatmap showing DEGs between the low- and high-risk groups. **(B)** Volcano plot showing DEGs between the low- and high-risk groups. **(C)** Kyoto Encyclopedia of Genes and Genomes (KEGG) pathway analysis. **(D-F)** Gene Ontology (Go) analysis.

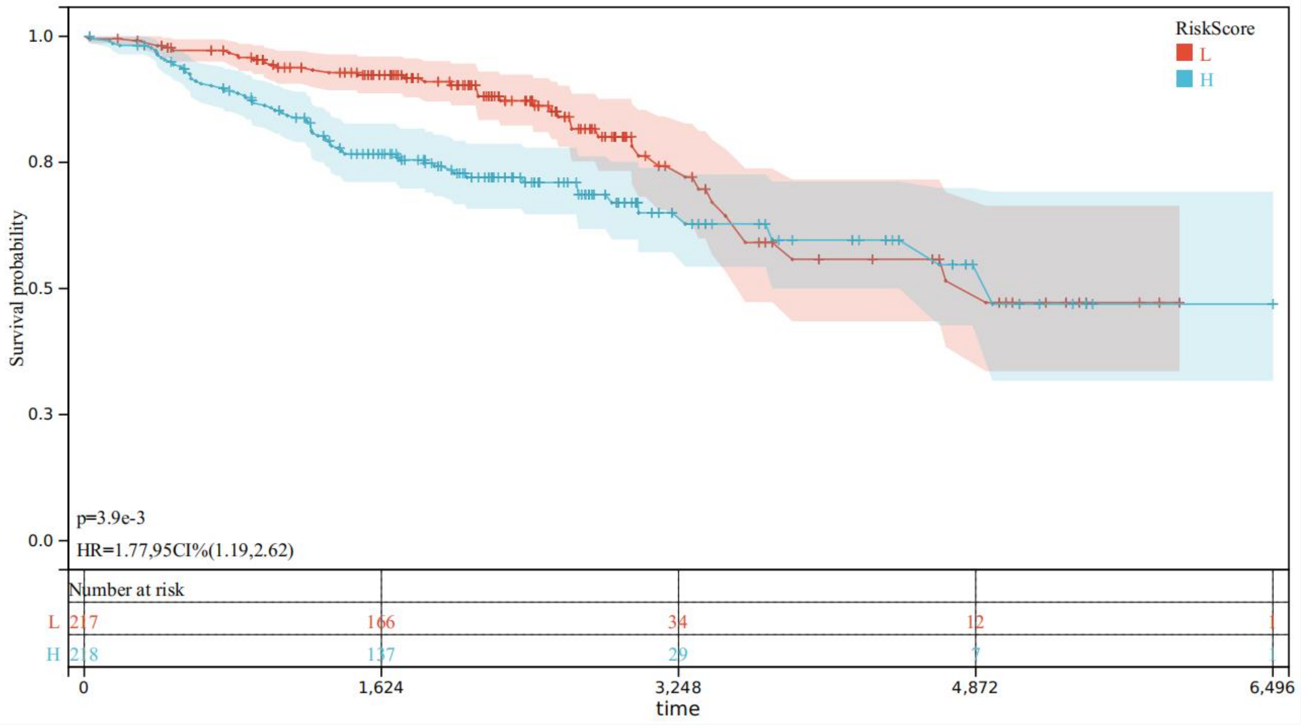

Supplementary Figure 9. Correlations of risk score and OS in the validation cohort.
